# Revisiting the rural and urban divide in hospital health information technology adoption: Evidence from 2023

**DOI:** 10.1111/jrh.70100

**Published:** 2025-12-10

**Authors:** Alice S. Yan, Teagan K. Maguire, Jie Chen

**Affiliations:** ^1^ Department of Health Policy and Management School of Public Health University of Maryland College Park Maryland USA; ^2^ Hospital And Public Health Interdisciplinary Research (HAPPY) Lab School of Public Health University of Maryland College Park Maryland USA; ^3^ University of Maryland Center on Aging School of Public Health University of Maryland College Park Maryland USA; ^4^ University of Maryland Center for SUNSHINE School of Public Health University of Maryland College Park Maryland USA

**Keywords:** health information technology, health information exchange, patient engagement, telehealth, rural health

## Abstract

**Purpose:**

We assessed the adoption of telehealth, patient engagement (PE), and health information exchange (HIE) functionalities among hospitals in 2023, comparing adoption rates between rural and urban hospitals.

**Methods:**

We used the linked 2023 American Hospital Association Annual Survey and Information Technology Survey data. We examined average adoption rates of eight telehealth, eight PE, and three HIE functionalities across metropolitan, micropolitan, and rural acute care hospitals. Multivariate regression models were used to assess differences in adoption, adjusting for hospital characteristics.

**Findings:**

Rural and urban disparities in hospital health information technology (HIT) adoption persist in 2023. After adjusting for hospital characteristics, rural and urban differences in the likelihood of adopting any treatment‐stage (e.g., psychiatric treatment and stroke care) or post‐discharge (e.g., remote patient monitoring for chronic care) telehealth were not significant. However, overall, rural hospitals adopted an average of 0.24 fewer telehealth services (*p* < 0.05) and 0.25 fewer PE capabilities (*p* < 0.05). They were also less likely to have any HIE capabilities, relative to their urban peers.

**Conclusions:**

Although overall adoption of hospital HIT has increased since 2018 and some rural and urban gaps have narrowed, disparities remain in 2023. Rural hospitals continue to lag behind in the adoption of telehealth, PE, and HIE functionalities. Future research should explore barriers to adoption among under‐resourced hospitals. Policy efforts must prioritize tailored strategies to support rural hospitals and promote broader access to HIT adoption nationwide.

## INTRODUCTION

Hospitals serve as major health care resources for underserved populations, particularly those in remote areas with poor health care infrastructure and a higher incidence of health disparities.^1^ Recent studies of hospital‐based health information technologies (HIT), such as telehealth‐assisted services, health information exchanges (HIEs), and patient engagement (PE) capabilities, have proven them to be effective at reducing urban/rural disparities in access to and quality of care.^1^, ^2^, ^3^ For example, the use of telehealth can promote timely delivery of care in rural counties lacking nearby health centers, while efficient HIE can minimize procedural redundancy and overall fragmentation of care within hospitals serving these communities.^1^, ^2^ Similarly, a digital platform that promotes PE can enhance the communication of health needs and coordination of care across multiple provider settings.^3^

Despite the potential of hospital‐based HIT for advancing equitable access to care in remote and underserved areas, the urban and rural digital divide persists, and consistent widespread HIT adoption is often hindered by various place‐based factors.^4^, ^5^, ^6^ For instance, rural counties face unique financial and resource‐based challenges, such as reimbursement for telehealth‐assisted services that is not commensurate with that of in‐person care, which has resulted in a shortage of providers willing to offer such services.^5^

In response to these challenges, a number of policy initiatives have aimed to close the urban and rural HIT adoption gap, particularly during the public health emergency triggered by the coronavirus pandemic. The pandemic exposed longstanding barriers to care in rural areas, while also revealing the potential of HIT‐based solutions to help bridge these gaps.^7^, ^8^ Federal efforts such as the Coronavirus Aid, Relief, and Economic Security Act expanded telehealth access in rural and medically underserved areas.^7^ In addition, waivers under the Coronavirus Preparedness and Response Supplemental Appropriations Act enabled Medicare providers to offer telehealth services to beneficiaries regardless of patient location.^7^ Several of these provisions remained in place through December 2024, leading to sustained increases in telehealth use in rural communities.^7^, ^8^ Building on these gains, recent evidence from 2023 suggests that critical access hospitals (CAHs), which comprised nearly two‐thirds of rural hospitals in 2023, are beginning to close the gap in adopting advanced PE tools and clinical data analytics capabilities.^9^, ^10^ Apathy et al. demonstrate that progress has been made, but also caution that CAHs may continue to struggle with adopting more advanced HIT functionalities, particularly interoperability standards such as Fast Healthcare Interoperability Resources (FHIR)‐based exchange.

However, despite these encouraging developments, the current state of hospital‐based HIT adoption across rural and urban settings remains uncertain. Chen et al. documented substantial challenges to telehealth implementation based on 2018 data, highlighting significant disparities between hospital types and geographic regions.^11^ Since that time, federal investments and pandemic‐era policy waivers may have contributed to narrowing these gaps, but it is unclear whether meaningful progress has been made or whether structural barriers continue to limit adoption, particularly in rural areas. To address this gap, our study examined 2023 hospital‐level adoption rates of three key HIT functionalities: telehealth, PE, and HIE, across metropolitan (metro), micropolitan (micro), and rural hospitals. We hypothesized that although overall adoption would likely increase, disparities between rural and urban hospitals would persist in 2023, even if somewhat reduced compared to 2018. In the discussion, we compare our findings to those reported by Chen et al. to assess progress over time and to identify areas where disparities remain.

## MATERIALS AND METHODS

### Data sources

We linked the 2023 American Hospital Association (AHA) Annual Survey and related Information Technology (IT) Survey.^12^, ^13^ Our study population included general medical and surgical (acute care) hospitals.

### Measurement

#### Outcomes

We evaluated hospital adoption of three categories of hospital‐based HIT functionalities: telehealth, PE, and HIE. To enable comparison with hospital adoption rates in 2018, we constructed these HIT adoption measures using definitions and methods consistent with Chen et al. The split of the telehealth measures into treatment‐stage and post‐discharge categories was based on prior literature.^14^

Telehealth measures were sourced from the AHA Annual Survey. We constructed six indices to measure adoption of telehealth services, including three binary indicators and three count indicators. The first binary indicator was for “treatment‐stage telehealth,” equaling 1 if a hospital reported using telehealth for one or more of the following: (1) consultation and office visits, (2) intensive care, (3) stroke care, (4) psychiatric and addiction treatment, and (5) other telehealth services, or 0 otherwise. The second binary indicator was for “post‐discharge telehealth,” equaling 1 if a hospital reported using telehealth for one or more of the following: (1) remote patient monitoring (RPM) post‐discharge, (2) RPM for chronic care, and (3) other RPM services, or 0 otherwise. The third binary indicator was for “overall telehealth,” equaling 1 if a hospital reported using telehealth for one or more of the eight listed services, and 0 otherwise. The corresponding count indicators represent the total number of telehealth services adopted in each category, ranging from 0 to 5 for “treatment‐stage telehealth,” 0 to 3 for “post‐discharge telehealth,” and 0 to 8 for “overall telehealth.”

PE measures were derived from the AHA IT Survey and captured the extent of patient access to and interaction with electronic health information. These measures included whether hospital patients could (1) view their medical record in their portal, (2) download their medical record from their portal, (3) import their medical record from an external provider into their portal, (4) transmit their medical record to an external provider from their portal, (5) submit patient‐generated data, (6) exchange secure messages with external providers, (7) view clinical notes in their portal, and (8) access their medical record using applications configured to meet Electronic Health Record (EHR) application programming interfaces specifications. We constructed a count indicator, ranging from 0 to 8, to represent the number of PE capabilities adopted by each hospital.

HIE measures were also obtained from the AHA IT Survey and included three binary indicators: (1) hospital ability to electronically query patient health data from external providers (“electronic data query capability”), (2) availability of patient health data to a hospital electronically from external providers (“electronic data availability”), and (3) hospital use of patient health data obtained electronically from external providers (“electronic data use”). Each measure was coded as 1 if the hospital reported having the capability and 0 otherwise. We also constructed a binary indicator for HIE adoption overall, equaling 1 if a hospital reported having one or more of the three listed capabilities, and 0 otherwise.

#### Exposures

Our independent variable of interest was hospital geographic location, measured using the AHA Annual Survey based on the US Office of Management and Budget's Core‐Based Statistical Areas (CBSAs).^15^ Hospitals were classified into three groups: metro, micro, and rural. A metro CBSA includes at least one urbanized area with a population of 50,000 or more, along with adjacent counties that demonstrate a high degree of social and economic integration with the core, as measured by commuting ties. A micro CBSA is comprised of urban clusters with total population between 10,000 and 49,999. Rural areas refer to all regions that fall outside of metro and micro CBSAs.

#### Covariates

These included hospital characteristics likely associated with HIT adoption, such as control type (not for profit, for profit, and government‐owned hospitals), bed size (small: <50 beds, medium: 50–99 beds, and large: ≥100 beds), teaching designation, and health system affiliation, in accordance with previous studies.^6^, ^11^

### Analytical strategy

We compared descriptive statistics for hospital HIT measures and other hospital characteristics by metro, micro, and rural location for the year 2023. Student's *t*‐tests were used differences across locations, with metro hospitals as the reference group. To visualize geographic variation in HIT adoption, we created a bar chart showing unadjusted hospital adoption rates for telehealth, PE, and HIE functionalities across the three location categories.

To assess the relationship between hospital geographic location and HIT adoption, we first employed univariate regression analyses, followed by multivariate models controlling for hospital characteristics. Specifically, logistic regressions were used for binary outcomes, including full/partial versus no adoption of treatment‐stage, post‐discharge, and overall telehealth services, and each of the three HIE adoption measures. To better capture the granularity of HIT adoption across hospitals, we also treated the number of adopted functionalities as a continuous variable. Ordinary least squares regressions were applied to estimate the number of treatment‐stage, post‐discharge, and overall telehealth services adopted, and the number of PE capabilities adopted. Since only 11 out of 1970 hospitals had not adopted PE, we focused on the number of PE functionalities adopted rather than a binary indicator. Marginal effects were reported for all models, and standard errors were clustered at the hospital level.

### Supplementary analyses

To assess the robustness of our findings, we conducted several sensitivity analyses. These included testing alternative model specifications, such as excluding/including hospital system affiliation as a covariate and using different definitions for key outcome measures. For example, given the unique role of CAH within rural communities, we explored patterns of HIT adoption within rural communities and the CAH designation unique to this setting, we conducted additional analyses comparing HIT adoption rates between rural CAHs and non‐CAHs. These results are presented in Appendices A and B.

All statistical analyses were performed using Stata MP, Version 18.^16^ Our study followed STROBE reporting guidelines and was exempt from IRB review with a waiver of informed consent, owing to the use of deidentified data per 45 CFR §46.

## RESULTS

Our final sample included 3004 hospitals for telehealth services, 1970 hospitals for PE capabilities, and 1936 hospitals for HIE capabilities, with rural hospitals representing approximately 20% of each sample. Table 1 showed that average adoption rates across all HIT functionalities were inversely associated with hospital rurality. The mean overall number of telehealth services adopted is 2.5 out of 8 (2.8 for metro hospitals, 2.3 for micro hospitals, and 1.8 for rural hospitals). The mean overall number of PE capabilities adopted is 6.9 out of 8 (7.1 for metro hospitals, 6.8 for micro hospitals, and 6.5 for rural hospitals).

**TABLE 1:** Hospital health information technology (HIT) adoption rates and other characteristics by urban/rural location (2023).

	Metropolitan hospitals			Micropolitan hospitals			Rural hospitals			Overall		
Hospital HIT measures	*n*	Mean	SD	*n*	Mean	SD	*n*	Mean	SD	*n*	Mean	SD
Telehealth measures												
Overall number of telehealth services adopted	1865	2.79	2.26	505	2.30***	1.92	640	1.80***	1.61	3010	2.50	2.12
Number of treatment‐stage telehealth services adopted	1865	2.06	1.48	505	1.81***	1.42	640	1.43***	1.23	3010	1.88	1.44
Number of post‐discharge telehealth services adopted	1865	0.73	1.09	505	0.49***	0.88	640	0.37***	0.72	3010	0.61	1.00
Overall telehealth service adoption (full/partial vs. none)	1865	82%	38%	505	80%	40%	640	78%**	42%	3010	81%	39%
Treatment‐stage telehealth service adoption (full/partial vs. none)	1865	81%	39%	505	80%	40%	640	75%***	43%	3010	80%	40%
Consultation/office visits	1865	62%	49%	505	66%	47%	640	64%	48%	3010	63%	48%
Stroke care	1865	58%	49%	505	45%***	50%	640	29%***	46%	3010	50%	50%
Other services	1865	31%	46%	505	30%	46%	640	26%*	44%	3010	29%	46%
Psychiatric/addiction treatment	1865	35%	48%	505	22%***	42%	640	17%***	38%	3010	29%	45%
Intensive care	1865	21%	40%	505	18%	38%	640	7%***	26%	3010	17%	38%
Post‐discharge telehealth service adoption (full/partial vs. none)	1865	37%	48%	505	28%***	45%	640	25%***	43%	3010	33%	47%
Remote patient monitoring for chronic care	1865	26%	44%	505	22%	42%	640	21%*	41%	3010	24%	43%
Other remote patient monitoring	1865	24%	43%	505	12%***	33%	640	8%***	27%	3010	19%	39%
Remote patient monitoring post‐discharge	1865	23%	42%	505	14%***	35%	640	7%***	26%	3010	18%	39%
PE measures												
Number of PE capabilities adopted	1262	7.09	1.28	330	6.81***	1.33	384	6.46***	1.56	1976	6.92	1.37
View medical record in portal	1291	99%	9%	341	100%	5%	395	100%	5%	2027	99%	8%
Download medical record from portal	1287	98%	13%	341	98%	15%	392	97%	17%	2020	98%	14%
Exchange secure messages with external provider	1286	95%	22%	340	93%	26%	392	93%	25%	2018	94%	23%
View clinical notes in portal	1289	97%	16%	340	96%	18%	393	91%***	29%	2022	96%	20%
Transmit medical record to external provider	1279	90%	30%	338	86%	35%	393	78%***	41%	2010	87%	34%
Access medical record using API‐configured apps	1289	92%	27%	339	85%***	36%	391	78%***	42%	2019	88%	33%
Submit patient‐generated data	1276	73%	44%	333	67%*	47%	392	57%***	50%	2001	69%	46%
Import medical record from external provider	1284	64%	48%	338	57%**	50%	392	51%***	50%	2014	60%	49%
HIE measures												
HIE capability adoption (full/partial vs. none)	1241	97%	16%	327	97%	18%	374	90%***	31%	1942	96%	20%
Electronic data query capability	1273	95%	22%	338	93%	26%	381	85%***	36%	1992	93%	26%
Electronic data use	1246	90%	30%	327	80%***	40%	377	72%***	45%	1950	85%	36%
Electronic data availability	1258	84%	36%	330	75%***	44%	379	64%***	48%	1967	79%	41%

Other Hospital Characteristics	*n*	Frequency	%	*n*	Frequency	%	*n*	Frequency	%	*n*	Frequency	%
Hospital control type												
Not for profit	1865	1392	74%	505	352***	69%	640	362***	56%	3010	2106	70%
For profit		235	13%		39***	8%		30***	5%		304	10%
Government		238	13%		114***	23%		248***	39%		600	20%
Hospital bed size												
Small: <50 beds	1865	328	18%	505	258***	51%	640	512***	80%	3010	1098	36%
Medium: 50–99 beds		206	11%		113***	22%		87***	14%		406	13%
Large: ≥100 beds		1331	71%		134***	27%		41***	6%		1506	51%
Hospital teaching designation												
Teaching hospital	1865	1245	67%	505	174***	34%	640	88***	14%	3010	1507	51%
Not a teaching hospital		620	33%		331***	66%		552***	86%		1503	49%
Hospital system affiliation												
Affiliated with a health system	1865	1524	82%	505	328***	65%	640	326***	51%	3010	2178	72%
Not affiliated with a health system		341	18%		177***	35%		314***	49%		832	28%

*Note*: The study population consists of general medical and surgical (acute care) hospitals. Data on hospital urban/rural location (Core‐Based Statistical Area [CBSA]), telehealth service adoption, and other hospital characteristics were sourced from the 2023 American Hospital Association (AHA) Annual Survey. Data on hospital patient engagement (PE) and health information exchange (HIE) capability adoption were sourced from the 2023 AHA Annual Information Technology (IT) Survey. Differences in sample sizes are due to varying response rates to the underlying survey questions regarding telehealth services and PE/HIE capabilities. Student's *t*‐tests were used for significance testing of differences across hospital locations, with metropolitan hospitals serving as the reference group.

* *p* < 0.05.

** *p* < 0.01.

*** *p*<0.001.

**FIGURE 1:**
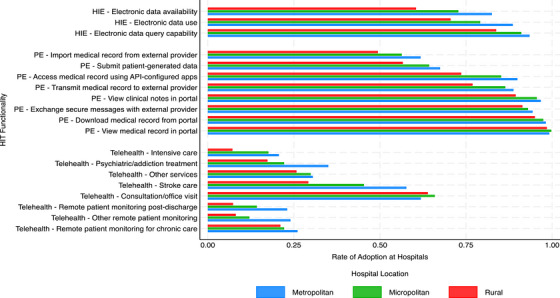
Bar chart of hospital health information technology (HIT) adoption rates by functionality and urban/rural location (2023). HIE, health information exchange; PE, patient engagement.

Consistent with Table 1, Figure 1 shows that among all HIT measures, telehealth had the lowest overall adoption rates across all geographies, whereas PE and HIE had higher rates. Only 7% of rural hospitals reported adopting RPM post‐discharge, compared to 23% of metro hospitals, a difference of 16 percentage points (*p* < 0.001). Among PE functionalities, the highest adoption rates were observed for the following: viewing medical records in the patient portal, downloading medical records, viewing clinical notes, and exchanging secure messages with external providers. These functionalities also showed the smallest rural‐urban adoption gaps.

Overall, rural hospitals reported lower adoption across nearly all HIT functionalities. The largest gaps were observed in telehealth for stroke care, RPM post‐discharge, other RPM services, intensive care, and psychiatric/addiction treatment. Gaps were also observed in electronic data availability and use. Interestingly, rural hospitals reported slightly higher adoption of telehealth for consultation and office visits compared to their metro counterparts.

Table 2 univariate analysis (Model 1) shows that rural hospitals were significantly less likely to adopt, or adopted fewer, HIT functionalities compared to metro hospitals, with micropolitan hospitals falling in between. After adjusting for hospital characteristics in multivariate models (Model 2), some of these differences were no longer statistically significant. Specifically, the differences for micropolitan hospitals were no longer significant. However, rural hospitals continued to adopt significantly fewer telehealth services overall, including 0.24 fewer services on average (*p* < 0.05), with 0.15 fewer treatment‐stage services (*p* < 0.05) and 0.10 fewer post‐discharge services (*p* < 0.05).

**TABLE 2:** Marginal effects of hospital urban/rural location on telehealth adoption (2023).

	Treatment‐stage telehealth measures				Post‐Discharge Telehealth Measures				Overall Telehealth Measures			
	Full/partial versus no service adoption		Number of services adopted		Full/partial vsersus no service adoption		Number of services adopted		Full/partial vsersus no service adoption		Number of services adopted	
	*n* = 3004		*n* = 3004		*n* = 3004		*n* = 3004		*n* = 3004		*n* = 3004	
	**ME**	**SE**	**ME**	**SE**	**ME**	**SE**	**ME**	**SE**	**ME**	**SE**	**ME**	**SE**
**Model 1: Univariate**												
Hospital geography (Ref. = Metropolitan)												
Micropolitan	−0.02	0.02	−0.25***	0.07	−0.09***	0.02	−0.25***	0.05	−0.02	0.02	−0.49***	0.10
Rural	−0.06**	0.02	−0.62***	0.06	−0.13***	0.02	−0.37***	0.04	−0.05**	0.02	−0.99***	0.08
**Model 2: Multivariate (Model 1 + other hospital characteristics)**												
Hospital geography (Ref. = Metropolitan)												
Micropolitan	0.02	0.02	0.04	0.08	−0.02	0.03	−0.07	0.05	0.02	0.02	−0.03	0.10
Rural	0.00	0.02	−0.15*	0.07	−0.01	0.03	−0.10*	0.05	0.01	0.02	−0.24*	0.10
Hospital control type (Ref. = Not for profit)												
For profit	−0.27***	0.03	−1.05***	0.07	−0.25***	0.02	−0.54***	0.04	−0.28***	0.03	−1.59***	0.09
Government	−0.06**	0.02	−0.14	0.07	−0.03	0.02	−0.02	0.05	−0.07***	0.02	−0.16	0.11
Hospital bed size (Ref. = Small: <50 beds)												
Medium: 50–99 beds	0.02	0.03	0.12	0.08	0.05	0.03	0.10*	0.05	0.02	0.02	0.22*	0.11
Large: ≥100 beds	0.10***	0.02	0.47***	0.07	0.15***	0.02	0.35***	0.05	0.09***	0.02	0.82***	0.10
Hospital teaching designation (Ref. = Not a teaching hospital)												
Teaching hospital	0.02	0.02	0.30***	0.06	0.07***	0.02	0.20***	0.04	0.03	0.02	0.49***	0.09
Hospital system affiliation (Ref. = Not affiliated with a health system)												
Affiliated with a health system	−0.01	0.02	0.20***	0.06	−0.04	0.02	−0.07	0.04	−0.02	0.02	0.13	0.09

*Note*: The study population consists of general medical and surgical (acute care) hospitals. Univariate regression analyses were run to estimate the association between hospital urban/rural location and telehealth adoption, and expanded into multivariate analyses controlling for other hospital characteristics. Logistic regressions were applied for the full/partial versus no service adoption outcomes, while OLS regressions were applied for the number of services adopted outcomes. Marginal effects (MEs) were reported for all models, and standard errors (SEs) were clustered at the hospital level. Data on hospital urban/rural location (Core‐Based Statistical Area [CBSA]), telehealth service adoption, and other hospital characteristics were sourced from the 2023 American Hospital Association (AHA) Annual Survey.

* *p* < 0.05.

** *p* < 0.01

*** *p* < 0.001.

Similar patterns were observed for PE and HIE measures, as shown in Table 3. After adjusting for hospital characteristics, rural hospitals adopted 0.25 fewer PE capabilities than metro hospitals on average (*p* < 0.05). They were also significantly less likely to have core HIE functionalities: 4 percentage points less likely to have electronic data query capabilities (*p* < 0.05), 8 percentage points less likely to report electronic data availability (*p* < 0.01), and 9 percentage points less likely to use electronic data from external providers (*p* < 0.001), compared to their urban counterparts.

**TABLE 3:** Marginal effects of hospital urban/rural location on patient engagement (PE)/health information exchange (HIE) adoption (2023).

	PE measures		HIE measures					
	Number of capabilities adopted		Electronic data query capability: Adoption versus no adoption		Electronic data availability: Adoption versus no adoption		Electronic data use: Adoption versus no adoption	
	*n* = 1970		*n* = 1936		*n* = 1936		*n* = 1936	
	ME	SE	ME	SE	ME	SE	ME	SE
**Model 1: Univariate**								
Hospital geography (Ref. = Metropolitan)								
Micropolitan	−0.28***	0.08	−0.02	0.02	−0.10***	0.03	−0.10***	0.02
Rural	−0.63***	0.09	−0.10***	0.02	−0.20***	0.03	−0.18***	0.02
**Model 2: Multivariate (Model 1 + other hospital characteristics)**								
Hospital geography (Ref. = Metropolitan)								
Micropolitan	−0.10	0.08	−0.01	0.02	−0.05	0.03	−0.06**	0.02
Rural	−0.25*	0.11	−0.04*	0.02	−0.08**	0.03	−0.09***	0.03
Hospital control type (Ref. = Not for profit)								
For profit	−1.61***	0.15	−0.27***	0.04	−0.17***	0.04	−0.20***	0.04
Government	−0.15	0.10	−0.02	0.01	−0.13***	0.03	−0.01	0.02
Hospital bed size (Ref. = Small: <50 beds)								
Medium: 50–99 beds	0.10	0.10	0.01	0.02	0.00	0.03	−0.01	0.02
Large: ≥100 beds	0.12	0.10	0.04*	0.02	0.01	0.03	−0.01	0.02
Hospital teaching designation (Ref. = Not a teaching hospital)								
Teaching hospital	0.14	0.07	0.01	0.01	0.01	0.02	0.02	0.02
Hospital system affiliation (Ref. = Not affiliated with a health system)								
Affiliated with a health system	0.88***	0.09	0.08***	0.02	0.23***	0.03	0.26***	0.03

*Note*: The study population consists of general medical and surgical (acute care) hospitals. Univariate regression analyses were run to estimate the association between hospital urban/rural location and PE/HIE adoption, and expanded into multivariate analyses controlling for other hospital characteristics. Logistic regressions were applied for all three HIE outcomes, while OLS regressions were applied for the number of PE capabilities adopted. Marginal effects (MEs) were reported for all models, and standard errors (SEs) were clustered at the hospital level. Data on hospital urban/rural location (Core‐Based Statistical Area [CBSA]) and other hospital characteristics were sourced from the 2023 American Hospital Association (AHA) Annual Survey. Data on hospital PE and HIE capability adoption were sourced from the 2023 AHA Annual IT Survey. Differences in sample sizes are due to varying response rates to the underlying survey questions regarding PE/HIE capabilities.

* *p* < 0.05.

** *p* < 0.01.

*** *p* < 0.001.

In addition to geographic disparities, several hospital characteristics were significantly associated with HIT adoption. For‐profit and government hospitals were generally less likely to adopt HIT than not‐for‐profit hospitals. Large hospitals (≥100 beds) and teaching hospitals reported greater use and adoption of telehealth services. System‐affiliated hospitals adopted more PE capabilities (ME = 0.88, *p* < 0.001) and were more likely to report all three HIE functionalities compared to nonaffiliated hospitals.

## DISCUSSION

Our findings indicate that a rural technology gap in HIT adoption persisted in 2023, with rural hospitals continuing to lag behind metro hospitals in telehealth, PE, and HIE capabilities. While hospital characteristics such as ownership, size, and teaching status explained part of this disparity, rural and urban differences remained significant after adjustment, highlighting persistent structural barriers to HIT adoption in rural settings.

These barriers are largely driven by two long‐standing challenges: limited broadband infrastructure and constrained financial capacity. Inadequate broadband access remains a defining obstacle for rural health systems and communities.^17^, ^18^, ^19^ As of 2021, approximately one‐third of rural residents still lacked high‐speed internet at home, limiting hospitals’ ability to deploy telehealth and health technologies.^17^, ^18^, ^19^, ^20^ Continued investment through federal, state, and public‐private partnerships will be essential to closing the broadband infrastructure gap.

Financial limitations also hinder rural hospitals’ ability to invest in technology.^21^ These hospitals typically serve smaller, publicly insured populations and operate with lower margins.^10^, ^21^, ^22^ In 2023, 19% of discharges at rural hospitals were covered by private insurance, relative to 24% at urban hospitals; at around this same time, private insurers paid hospitals 254% of Medicare rates, on average.^23^, ^24^ Because rural hospitals depend more heavily on Medicare and Medicaid, they are especially vulnerable to reimbursement changes. Nearly half of rural Medicare beneficiaries are now enrolled in MA plans, representing a dramatic shift away from traditional Medicare, and with uncertain implications, as some hospital administrators are sounding the alarm that MA plans have lower reimbursement rates and greater administrative burden compared to traditional Medicare.^25^ Looking ahead, potential Medicaid cuts could further threaten the financial sustainability of rural hospitals.^25^, ^26^ Together, these constraints help explain why rural hospitals continue to lag in breadth of HIT adoption, even after adjusting for observable characteristics. The following sections explore how these challenges play out across specific domains of telehealth, PE, and HIE.

Specifically for telehealth, RPM post‐discharge and RPM for chronic care were among the least adopted hospital HIT capabilities, with significantly lower adoption by rural hospitals. Prior research on rural readiness for RPM highlights two primary challenges: infrastructure limitations, particularly inadequate broadband connectivity, and low digital literacy among patients.^17^, ^27^ Yet, national use of RPM is expanding rapidly. Traditional Medicare spending on RPM increased from $6.8 million in 2019 to $194.5 million in 2023.^28^, ^29^ Despite this growth, rural residents use RPM less often than their urban counterparts, even though they stand to benefit substantially from its expansion.^28^, ^29^ Addressing both infrastructure and digital health literacy gaps is critical in preventing a widening rural‐urban divide as RPM becomes more central to chronic disease management and post‐acute care. A recent PEW Research Center study found that rural residents consistently report lower levels of technology ownership than more urban residents, underscoring the need for tailored rural programs that strengthen digital health literacy.^20^

Substantial gaps also persisted in specialized telehealth services. Rural hospitals are far less likely to offer telehealth for stroke care, tele‐intensivist services, and tele‐psychiatry or addiction treatment than metro hospitals. These gaps may partly reflect underlying differences between rural and urban hospital service line capacity. For example, in 2019, only 19% of rural emergency departments (EDs) offered any level of stroke center, compared to 46% of EDs nationally.^30^ Similarly, Ellison et al. found urban hospitals were nearly twice as likely as rural hospitals offer psychiatric services, and that rural hospitals were 1.5 times more likely to transfer patients requiring psychiatric admission.^31^ Thus, the gap may be demonstrating locally lacking service lines. It may also be signaling wider need and opportunity for provider‐to‐provider telehealth in specialty areas to alleviate disparities in access to care for rural patients.

Rural hospitals also lag modestly in the number of PE functionalities adopted (0.25 fewer on average), yet overall PE adoption increased substantially since 2018. Nearly all hospitals now provide core patient portal features, and in some areas, such as portals that allow patients to view their medical records, rural hospitals outmatch metro peers in our descriptive analysis. Emerging scholarship emphasizes next‐generation priorities including improving portal data accuracy, developing standardized management protocols, integrating patient‐driven data and feedback mechanisms, as well as increasing portal use.^32^ However, our study illustrates differences in the breadth of PE capabilities between rural and metro hospitals; as policymakers and administrators look beyond adoption to next‐generation priorities, it will be critical to ensure that rural patients benefit from the same degree of PE functionalities that metro patients do. Achieving this will require focused efforts to promote digital literacy, integrate portals into care workflows, and monitor how patients from rural communities engage with portal tools.^18^

In the HIE domain, rural hospitals in 2023 were still significantly less likely to have electronic query capabilities, report electronic data availability, and use data from external provider. Encouragingly, HIE adoption gaps narrowed since Chen et al. Progress in rural HIE adoption likely reflects improved interoperability, while previous literature identifies common barriers to HIE as lack of resources, interoperability challenges, computer illiteracy, lack of trust, privacy concerns, and challenges of overall use.^33^, ^34^ Strengthening shared networks, providing technical assistance, standardizing HIE across various HIE systems, and incentivizing optimization of HIE could continue to improve data sharing and interoperability, as well as continue to close adoption gaps between rural and urban geographies.^33^, ^34^

The study showed that although HIT adoption gaps between urban and rural hospitals persisted in 2023, disparities narrowed compared to 2018. For example, the telehealth for intensive care adoption gap narrowed from a 35 percentage point gap in 2018 to a 14 percentage point gap in 2023.^11^ Similarly, the gap between rural and metro hospitals in electronic data query capability declined from 24 percentage points in 2018 to 10 percentage points in 2023.^11^ In contrast to 2018, when rural hospitals lagged behind metropolitan hospitals by 3 and 2 percentage points, respectively, telehealth for consultations and office visits and patient portals that allow patients to view their medical records now have higher adoption rates among rural hospitals in 2023.^11^

### Policy implications

#### Economic forces shaping adoption

Broader market dynamics have shaped hospital HIT adoption. While HIT adoption has increased among surviving rural hospitals, the total number of rural hospitals has declined, which may obscure broader system‐level disparities. Since 2010, 150 rural hospitals have closed, including 64 closures between 2018 and 2023, and a net reduction of 50 rural hospitals occurred between 2017 and 2023.^22^, ^35^ Those that closed were often the most financially vulnerable and the least able to invest in infrastructure, including HIT.^22^, ^35^ Thus, rising HIT adoption among surviving rural hospitals may in part reflect a selective retention effect, where only the most capable of sustaining innovation (including investment in technology) remain.^36^, ^37^

At the same time, hospital consolidation and vertical integration are accelerating: between 2018 and 2023, 428 hospital and health system merger and acquisition (M&A) transactions were announced.^38^, ^39^ Technology is increasingly a strategic asset, making robust HIT infrastructure a strategic factor in M&A decisions.^40^ As a result, increased HIT adoption may also be driven by market pressures to remain competitive and attractive for partnerships. Further, we suspect there may be a bidirectional effect, whereby rural hospitals that become affiliated with larger health systems also then benefit from technology influx/investment.

### Federal investments and the path forward

Recent federal initiatives have played a critical role in narrowing urban–rural disparities in HIT. In 2018, the Centers for Medicare & Medicaid Services (CMS) launched its Rural Health Strategy, which identified telemedicine expansion as a core objective, and in 2020, the Department of Health and Human Services released its Rural Action Plan, which emphasized leveraging technology and innovation to improve rural health.^41^, ^42^ Long‐standing Federal Communications Commission programs, initiatives by the United States Department of Agriculture, and the 2021 Infrastructure Investment and Jobs Act have all helped expand and subsidize broadband infrastructure and high‐capacity telecommunications networks for rural hospitals and clinics.^43^, ^44^, ^45^ Collectively, these efforts have contributed to narrowing the rural–urban technology gap and improving hospital HIT readiness.

More recently, the *Rural Health Transformation Program* announced by CMS in 2025 signaled a renewed investment of over $50 billion over 5 years to strengthen rural health systems through HIT modernization, workforce development, and digital resilience.^46^ Together, these initiatives signal a sustained federal commitment to advancing digital health equity in rural America, and to rural American health.

However, adoption alone is not enough. Ensuring that these technologies translate into equitable use remains a critical challenge. Sustaining progress will require coordinated policy action, including investment in broadband infrastructure, reimbursement parity for telehealth services, support for shared HIT networks, workforce development, and incentives that reward both adoption and equitable use. Continued federal and state commitment to equity‐driven design, technical support, and rural‐targeted funding will be essential to ensure that advances in HIT benefit all communities, not just those with the greatest resources.

### Hospital adoption versus patient access

Finally, it is important to recognize that parity in hospital HIT adoption does not necessarily translate into equity in patient use. Disparities often persist in how patients access and engage with these technologies. For example, prior research shows that rural residents remain less likely than their urban counterparts to use patient portals, and that active portal users are disproportionately affluent, middle‐aged white women.^47^, ^48^, ^49^, ^50^ Moreover, rural residents are also less likely to be offered access to a portal by their health care providers. These patterns highlight the need to move beyond adoption rates as the sole success metrics and to evaluate meaningful and equitable use at the patient level.^51^, ^52^ Health care disparities must be measured not only by institutional readiness, but also by actual utilization across diverse populations.

### Limitations

Our study has several limitations. First, all regression estimates are interpreted as associational rather than causal, given the study was cross‐sectional in nature. Second, recall bias is a risk given the AHA Annual Survey and IT Survey data are self‐reported, as is non‐response bias given the low response rate to the HIT questions in both surveys.^53^, ^54^ Third, we examined hospital adoption rates of HIT, which may not equate to actual utilization. Future studies should explore the relationship between HIT implementation, actual usage patterns, and outcomes such as care quality, coordination, and patient health. Fourth, though we acknowledge the potentially significant influence of different EHR vendors and systems on hospital adoption of various HIT functionalities, we were unable to control for these characteristics in our analyses, given the data limitation. Finally, we did not evaluate differences by rural hospital designation (i.e., CAH vs. non‐CAH) beyond the limited analyses in Appendices A and B. Additional research on differences in HIT adoption between CAHs and non‐CAHs would be of great value, given CAHs comprise a large majority of rural hospitals.^10^

## CONCLUSION

While rural and urban disparities in hospital HIT adoption persist, overall adoption rates across all major functionalities have improved from 2018 to 2023. Encouragingly, several adoption gaps have also narrowed, such as in the provision of telehealth for consultations and office visits in 2023, suggesting promising implementation and potential impact for rural populations. However, gaps remain in key areas such as telehealth for post‐discharge purposes (RPM for chronic care, other RPM, and RPM post‐discharge). Future research should explore the characteristics of hospitals that remain non‐adopters of HIT and examine the structural and policy barriers that they face. Policy efforts should prioritize tailored solutions that address the unique challenges of rural hospitals, to ensure that all communities can benefit equitably from HIT‐enhanced care and to further bridge the digital divide.

## CONFLICT OF INTEREST STATEMENT

The authors declare no conflicts of interest.

## Data Availability

The American Hospital Association Annual Survey and Information Technology Survey data are restricted by a data use agreement, and cannot be made available.

## APPENDIX A

**TABLE 1A:** Health information technology (HIT) Adoption Rates and Other Characteristics of Rural Hospitals by critical access hospital (CAH) Designation (2023).

	Rural CAH designation			No rural CAH designation			Overall			
Hospital HIT measures	*n*	Mean	SD	*n*	Mean	SD	*n*	Mean	SD	*p*
**Telehealth measures**										
Overall number of telehealth services adopted	509	1.76	1.59	131	1.97	1.65	640	1.80	1.61	
Number of treatment‐stage telehealth services adopted	509	1.40	1.21	131	1.58	1.31	640	1.43	1.23	
Number of post‐discharge telehealth services adopted	509	0.36	0.70	131	0.39	0.79	640	0.37	0.72	
Overall telehealth service adoption (full/partial vs. none)	509	77%	42%	131	81%	39%	640	78%	42%	
Treatment‐stage telehealth service adoption (full/partial vs. none)	509	74%	44%	131	79%	41%	640	75%	43%	
Consultation/office visits	509	64%	48%	131	64%	48%	640	64%	48%	
Stroke care	509	27%	44%	131	37%	49%	640	29%	46%	^*^
Other services	509	27%	44%	131	22%	42%	640	26%	44%	
Psychiatric/addiction treatment	509	15%	36%	131	27%	44%	640	17%	38%	^***^
Intensive care	509	7%	26%	131	8%	27%	640	7%	26%	
Post‐discharge telehealth service adoption (full/partial vs. none)	509	25%	43%	131	24%	43%	640	25%	43%	
Remote patient monitoring for chronic care	509	21%	41%	131	21%	41%	640	21%	41%	
Other remote patient monitoring	509	8%	28%	131	7%	25%	640	8%	27%	
Remote patient monitoring post‐discharge	509	6%	25%	131	11%	31%	640	7%	26%	
**PE measures**										
Number of PE capabilities adopted	315	6.48	1.56	69	6.38	1.55	384	6.46	1.56	
View medical record in portal	325	100%	0%	70	99%	12%	395	100%	5%	^*^
Download medical record from portal	322	97%	17%	70	97%	17%	392	97%	17%	
Exchange secure messages with external provider	322	94%	24%	70	90%	30%	392	93%	25%	
View clinical notes in portal	323	91%	28%	70	90%	30%	393	91%	29%	
Transmit medical record to external provider	324	78%	41%	69	78%	42%	393	78%	41%	
Access medical record using API‐configured apps	321	77%	42%	70	81%	39%	391	78%	42%	
Submit patient‐generated data	322	56%	50%	70	63%	49%	392	57%	50%	
Import medical record from external provider	322	52%	50%	70	41%	50%	392	51%	50%	
**HIE measures**										
HIE capability adoption (full/partial vs. none)	307	90%	30%	67	87%	34%	374	90%	31%	
Electronic data query capability	312	85%	36%	69	84%	37%	381	85%	36%	
Electronic data use	310	75%	43%	67	58%	50%	377	72%	45%	^***^
Electronic data availability	311	65%	48%	68	59%	50%	379	64%	48%	

Other hospital characteristics	*n*	Frequency	%	*n*	Frequency	%	*n*	Frequency	%	
Hospital control type										
Nongovernment	509	302	59%	131	90	69%	640	392	61%	
Government		207	41%		41	31%		248	39%	
Hospital bed size										
Small: <50 beds	509	436	85%	131	76	58%	640	512	80%	^***^
Medium: 50–99 beds		55	11%		32	24%		87	14%	
Large: > = 100 beds		18	4%		23	18%		41	6%	
Hospital teaching designation										
Teaching hospital	509	52	10%	131	36	27%	640	88	14%	^***^
Not a teaching hospital		457	90%		95	73%		552	86%	
Hospital system affiliation										
Affiliated with a health system	509	248	49%	131	78	60%	640	326	51%	^*^
Not affiliated with a health system		261	51%		53	40%		314	49%	

*Note*: The study population consists of general medical and surgical (acute care) hospitals. Data on hospital urban/rural location (Core‐Based Statistical Area [CBSA]), CAH designation, telehealth service adoption, and other hospital characteristics were sourced from the 2023 American Hospital Association (AHA) Annual Survey. Data on hospital PE and HIE capability adoption were sourced from the 2023 AHA Annual Information Technology (IT) Survey. Differences in sample sizes are due to varying response rates to the underlying survey questions regarding telehealth services and patient engagement (PE)/health information exchange (HIE) capabilities. Analysis of variances and covariances (for continuous variables) and Pearson's chi‐square tests (for categorical variables) were used for significance testing of differences between rural CAHs and non‐CAHs.

**p*<0.05.

***p*<0.01.

****p*<0.001.

## APPENDIX B

**TABLE 1B:** Marginal Effects of Hospital Urban/Rural Location and Rural critical access hospital (CAH) Designation on Health information technology (HIT) Adoption (2023).

	Telehealth measures				PE measures		HIE measures					
	Full/partial versus no service adoption		Number of services adopted		Number of capabilities adopted		Electronic data query capability: Adoption versus no adoption		Electronic data availability: Adoption versus no adoption		Electronic data use: Adoption versus no adoption	
	*n* = 3004		*n* = 3004		*n* = 1970		*n* = 1936		*n* = 1936		*n* = 1936	
	ME	SE	ME	SE	ME	SE	ME	SE	ME	SE	ME	SE
**Model 1: Multivariate**												
Hospital geography (Ref. = Metropolitan)												
Micropolitan	0.01	0.02	−0.03	0.10	−0.10	0.08	−0.01	0.02	−0.05	0.03	−0.06^*^	0.02
Rural—Non‐CAH	0.04	0.03	−0.19	0.15	−0.32	0.18	−0.04	0.03	−0.10^*^	0.05	−0.21^***^	0.05
Rural—CAH	0.00	0.02	−0.26^*^	0.11	−0.24^*^	0.12	−0.03	0.02	−0.08^*^	0.03	−0.06^*^	0.03
Hospital control type (Ref. = Not for profit)												
For profit	−0.28^***^	0.03	−1.59^***^	0.09	−1.60^***^	0.15	−0.27^***^	0.04	−0.17^***^	0.04	−0.19^***^	0.04
Government	−0.07^***^	0.02	−0.16	0.11	−0.15	0.10	−0.02	0.01	−0.13^***^	0.03	−0.01	0.02
Hospital bed size (Ref. = Small: <50 beds)												
Medium: 50–99 beds	0.01	0.02	0.22*	0.11	0.11	0.11	0.01	0.02	0.00	0.03	−0.00	0.02
Large: > = 100 beds	0.09^***^	0.02	0.82^***^	0.10	0.13	0.10	0.04^*^	0.02	0.01	0.03	0.00	0.02
Hospital teaching designation (Ref. = Not a teaching hospital)												
Teaching hospital	0.03	0.02	0.49^***^	0.09	0.14	0.07	0.01	0.01	0.02	0.02	0.02	0.02
Hospital system affiliation (Ref. = Not affiliated with a health system)												
Affiliated with a health system	−0.02	0.02	0.13	0.09	0.88^***^	0.09	0.08^***^	0.02	0.23^***^	0.03	0.26^***^	0.03

*Note*: The study population consists of general medical and surgical (acute care) hospitals. Multivariate regression analyses were run to estimate the association between hospital urban/rural location and HIT adoption while controlling for other hospital characteristics. Logistic regressions were applied for full/partial versus no telehealth service adoption and all three health information exchange (HIE) outcomes, while OLS regressions were applied for number of telehealth services and PE capabilities adopted. Marginal effects (MEs) were reported for all models, and standard errors (SEs) were clustered at the hospital level. Data on hospital urban/rural location (Core‐Based Statistical Area [CBSA]), CAH designation, telehealth service adoption, and other hospital characteristics were sourced from the 2023 American Hospital Association (AHA) Annual Survey. Data on hospital patient engagement (PE) and HIE capability adoption were sourced from the 2023 AHA Annual Information Technology (IT) Survey. Differences in sample sizes are due to varying response rates to the underlying survey questions regarding telehealth services and PE/HIE capabilities.

* *p* <0.05.

** *p* < 0.01.

*** *p* < 0.001.
